# Interactive Learning System for Learning Calculus

**DOI:** 10.12688/f1000research.73595.2

**Published:** 2024-03-11

**Authors:** Md Asifur Rahman, Lew Sook Ling, Ooi Shih Yin

**Affiliations:** 1 Faculty of Information Science and Technology, Multimedia University, Ayer Keroh, Melaka, 75450, Malaysia

**Keywords:** Interactive learning system, augmented reality, learning experience, learning performance

## Abstract

**Background:**

IT tools has brought a new perspective to collaborative learning where students do not just sit in a chair and swallow lecture content but instead participate in creating and sharing knowledge. However, calculus learning augmented reality application has limitation in promoting a human collaboration in learning.

**Purpose:**

This research develops an interactive application for learning calculus that promotes human-system interaction via augmented reality (AR) and human-human interaction through chat functions. The study examines the effect of both interactivities on learning experience and how that learning experience affects the performance of learning.

**Methods:**

The research adopted a quasi-experimental study design and pre-post test data analysis to evaluate the effect of interactivities on learning experience and consequently the effect of learning experience on learning performance. The subjects were exposed to the developed application for learning the calculus chapter “Solid of Revolution” in a controlled environment. The study validated its research framework through partial least squares path modelling and tested three hypotheses via pre-and post-test evaluation.

**Conclusions:**

The results found that both interactivities affect learning experience positively; human-human interactivity has a higher impact than the human-system interactivity. It was also found that learning performance as part of the learning experience increased from pre-test to post-test.

## Introduction

In traditional classroom environments, there exists a problem with student participation. ^1^ It is also stated that the traditional method is inefficient as it is mostly spoon-feeding, and students’ analytical processes are absent due to a lack of peer interaction. ^2^ Although some studies have addressed the issues of student interaction with the system there is still a lack of teacher-student or student-student interaction in interactive learning systems. ^1^^–^^3^ Many systems have promoted a single type of interactivity. ^4^^–^^10^

Calculus is one of the core subjects in computer science studies. Malaysian students pursuing diplomas and degrees in Information Technology take calculus subjects that include differentiation, and integration. Application of integration is one of the chapters in Calculus that requires spatial visualization and the 2D medium of the traditional classroom does not provide a proper solution. ^11^^–^^13^

In understanding the revolution of solids around an axis, visualization of the solid in-question is very important. Spatially related 3D images cannot be drawn on a 2D board or projected over a computer. These hinder students in visualizing the solid and result in difficulties in conceptualizing the concept. After consulting with the subject teacher on what the student struggle most in learning calculus, it was suggested that students face difficulties in understanding solid of revolution through traditional classroom method as it requires 3D visualization. Although researchers have implemented AR to assist in visualization, they are mainly focused on human-system interaction, either through haptic or non-haptic interaction. ^14^^–^^17^ Besides, agility is another issue that some systems could not address as they are desktop-based systems. ^17^^–^^19^ Under the theory of learner constructing their knowledge Lev Vygotsky theorized that learning occurs through personalization and socialization. ^20^ So it is crucial to provide the function to interact among the learners. Unfortunately, majority of the AR learning application only focused on providing content visualization resulting in not a holistic application in learning that promote both type of interaction. ^14^^,^^15^^,^^21^^,^^22^ As such, developing an interactive AR application that facilitates both human-human and human-system interactivities and assists in conceptualizing and understanding math better is needed.

Interactive technology has made learning more personalized and kept students engaged through various applications. ^23^ This paper also suggested that interactive technology can make learning more active, and intensive for students where students can communicate easily with each-others and also with the teacher. ^23^ Recently implementation of Augmented Reality (AR) in pedagogy has been adopted by many researchers where the emphasis has mainly been kept on 3D visualization. Many have implemented AR in geometry for school students ^14^^,^^19^^,^^24^ while some others implemented it for calculus. ^12^^,^^13^ In both cases, the authors have focused on the immersive learning experience through AR but no human-human interaction function was provided.

This research aims to develops an augmented reality application that promotes interaction and spatial visualization for learning calculus and evaluates the effect of the interactive learning system on the performance of learning.

## Literature review

Implementation of interactive learning systems in class to engage students in learning has become a common focus in the pedagogical arena. A system that promotes interpersonal interaction and provides a sense of others’ presence can be considered as an interactive system. ^4^

### Interactivity

In an attempt to increase class interactivity different types of technologies have been implemented in class. These can be categorized as synchronous and asynchronous mediums of interaction. ^25^ Synchronous is used as a medium of interaction during class specially in carrying out in class discussion, whereas asynchronous is for facilitating learning remotely that may happen after the class. Interactive whiteboards, ^2^ and virtual-reality-based systems for learning language ^4^ are used in synchronous learning. AR-based interactive book ^5^ is a tool used in asynchronous learning. On the other hand, interactivity can also be categorized as human-system and human-human, as technologies are allowing both in a learning environment. ^3^ The framework for interactive learning application is developed based on these two types of interactions. Human-human interactivity is further divided into teacher-student and student-student interactivity. Past studies in Table 1 shows promoted interactivities by different studies. These studies focused primarily on human-system interactivity while a few provided human-human interactivities.

**Table 1.** Interactivities of different studies.

No	Study	Teacher-Student	Student-Student	Interactive system
1	^1^	✓	✓	×
2	^2^	✓	✓	✓
3	^4^	×	×	✓
4	^10^	×	×	✓
5	^5^	×	×	✓
6	^6^	×	×	✓
7	^49^	×	✓	✓
8	^9^	×	×	✓
9	^3^	✓	✓	✓
10	^7^	×	×	✓
11	^47^	✓	×	✓
12	^8^	×	×	✓

AR has become a trending technology in the pedagogical sector to provide students with an immersive experience. AR provides a real and virtual experience together to allow users real-time interaction. ^16^ 3D representation of a virtual object in a real environment can make learning more engaging as it provides a visual learning experience. This can be helpful to the majority of students as studies have found that there are more visual learners compared to auditory or kinesthetics learners. ^26^ Besides, haptic interaction happens when users are allowed to interact with the 3D object through AR technology ^27^ which also address kinesthetics learners as they learn through physical involvement. Past studies in Table 2 depicts the interactivities promoted by different AR studies.

**Table 2.** AR interactivities of different studies.

No	Study	Teacher-Student	Student-Student	Interactive book/system	Mobility
1	^16^	×	×	✓	✓
2	^27^	×	×	✓	✓
3	^14^	×	×	✓	✓
4	^18^	×	×	✓	×
5	^19^	×	×	✓	×
6	^17^	×	×	✓	×
7	^15^	×	×	✓	✓
8	^21^	×	×	✓	✓

From the past studies depicted in Table 2, five papers adopted mobility in their AR system except the three that were desktop-based. Table 2 depicts that most systems solely focused on interactivity through AR and not implementing any human-human interactivity.

### Learning Experience

Learning experience can be defined as the experience a learner goes through while learning content set by an institution. ^28^ Including active engagement and collaboration as a part of it affects learning performances. ^29^^,^^30^ Furthermore, implementation of technology in class affects the learning experience positively. ^31^

## Methods

### Research Design and Participants

This study conducted a pre-assessment evaluation of the respondents’ knowledge, skills, or understanding in the AR area related to the system before they started using it. ^32^ One of the numerous forms of quasi-experimental design is pre- and post-test research. “Quasi” refers to something that resembles experimental study. Studies evaluating a curriculum for education, a treatment system or a simulation training commonly apply pre- and post-test evaluation. ^33^ Since this study is to compare students’ learning experiences and academic performance before and after using the AR system, a pre- and post-test quasi-experimental research method was chosen.

Convenience sampling is a nonprobability or non-random sampling in which it is able to conveniently reach and recruit members of the target population for the study. ^34^ Usually, convenience samples of university students are used in academic surveys. ^35^ Since the respondents were students from a Malaysian university taking Calculus subject, a non-probability convenience sampling method was used for this study.

Sample size calculation was done by following the 10 times rule, where the sample size is required to be 10 times larger than the maximum number of structural paths directed towards a latent variable. ^36^ As such the minimum sample for this study is supposed to be larger than 20 as two paths are directed towards the latent variable. The sample size for this research was 59, but after eliminating missing data and straight-line answers the data was analyzed from 55 respondents.

Quantitative research designs include quasi-experiments, true experiments, causal-comparative research, surveys research and experiments research. Quantitative research is likely to test theories by investigating the relationships among variables. ^37^ Therefore, the primary research method for this study was quantitative.

Structural equation modeling (SEM) combines multiple regression and factor analysis. It is employed to examine the relationships between a group of observable variables and latent concepts. There are two main types of the SEM. In this study, instead of using the traditional Covariance-Based-SEM (CB-SEM), Partial Least Squares-SEM (PLS-SEM). PLS-SEM is chosen to analyze the data based on its capability to handle complex models and small sample sizes. Data analysis using PLS-SEM was employed by defining clear research objectives and hypotheses, data preparation, structural model analysis, hypotheses testing, structural model assessment and predictive accuracy, effect size and relevance evaluation. ^36^ PLS-SEM is often chosen for longitudinal studies due to its effectiveness in handling small sample sizes, which are common in these studies, especially when compared to cross-sectional studies. ^36^

**Figure 1.**
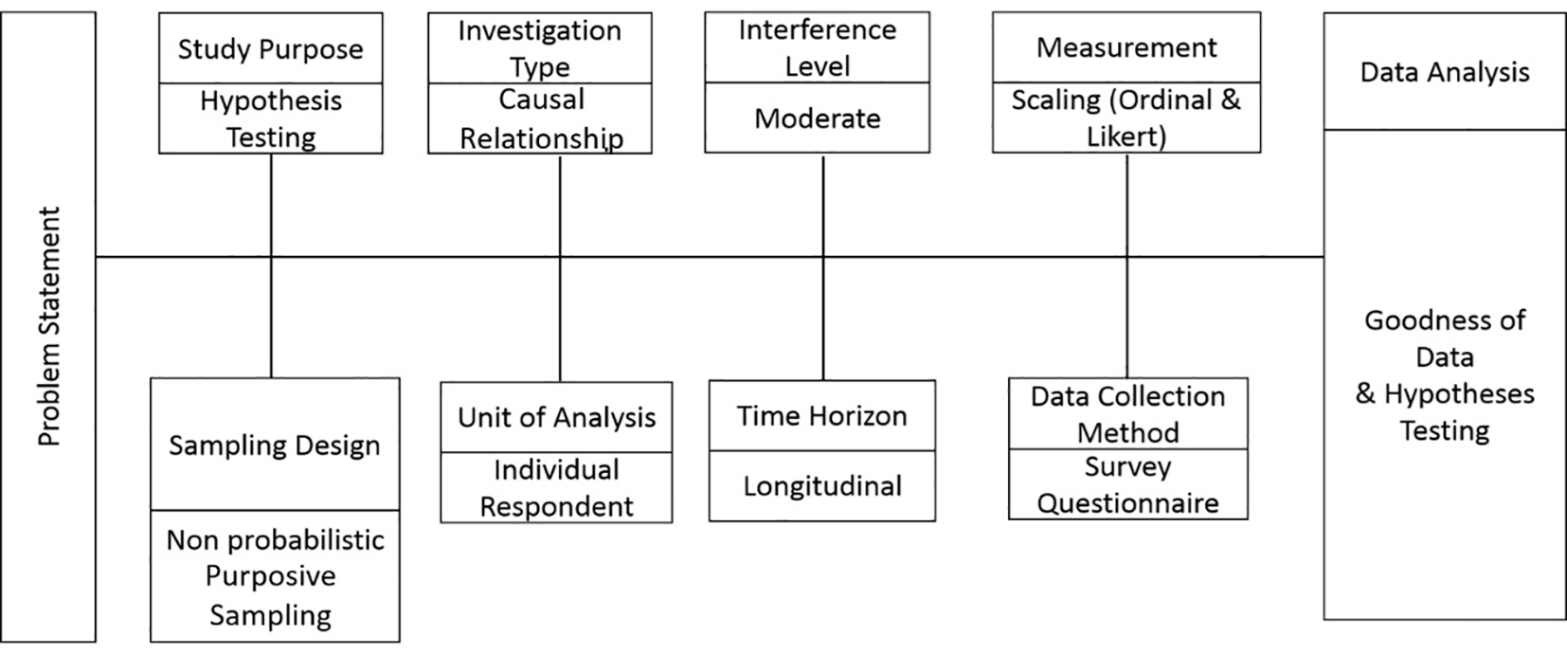
Research design.

Figure 1 illustrates the research design of this study.

### Research framework

**Figure 2.**
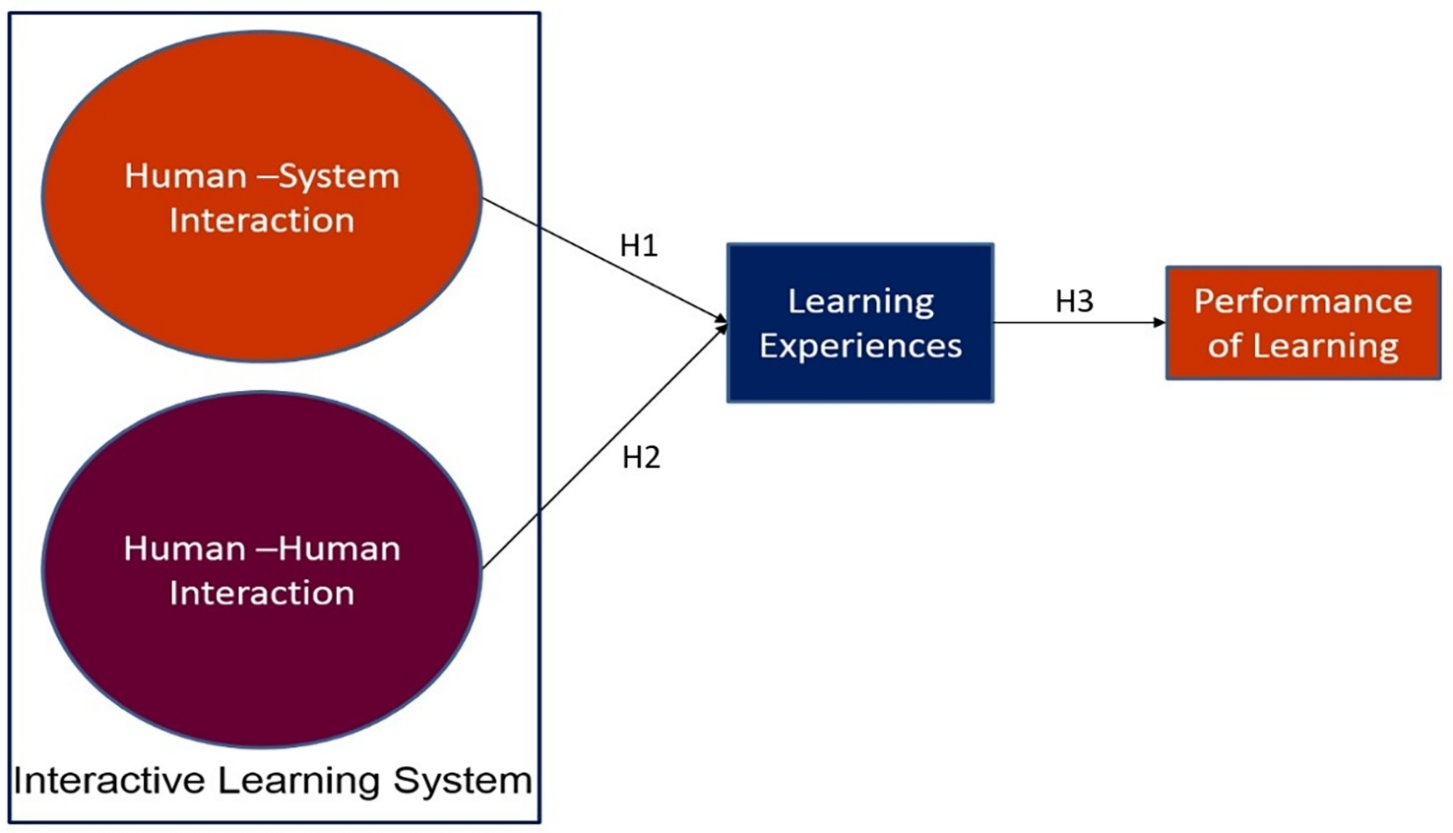
Research framework.

This research has identified two types of interactivities, human-human and human-system. Figure 2 depicts the research framework of this study.

An interactive learning system based on AR was developed. Human-system and human-human interactivities were included as part of application functions. Human-system interaction was promoted by haptic interaction based on marker-based AR technology whereas human-human interactivity was implemented through a function of discussion platform. The framework was developed to find how these two interactivities affect the learning experience, the first dependent variable, and lastly how the learning experience incurred by the promoted interactivities affects students’ academic performance.

This study hypothesizes:

H1: Human-system interaction is positively associated with students’ learning experience.

H2: Human-human interaction is positively associated with students’ learning experience.

H3: Improvement of learning experience improves the performance of learning.

### Survey Design and Procedures

This research was conducted into three phases. In the first phase, data were collected from students through a pre-questionnaire and a quiz before using the application, in the second phase, they used the application and explored AR, and lastly, in the third phase, post-test data were collected through same questionnaire.

**Figure 3.**
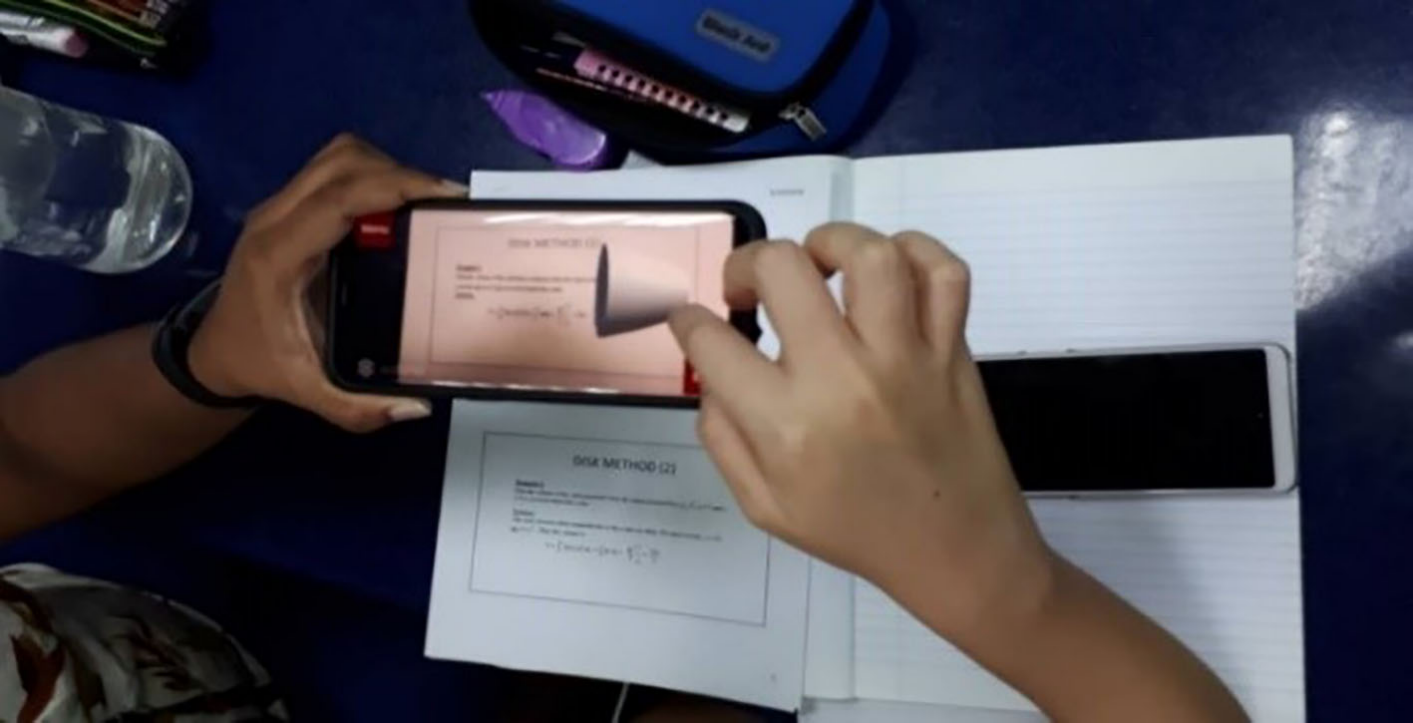
Implementation of AR system in class.

Figure 3 shows students exploring the AR function of the application.

The survey questionnaire was divided into three segments. Section (A) was demographic questions; section (B) was for evaluating the two independent variables, section (C) for measuring the learning performance.

**Section A** comprises of six questions regarding participants’ general information that included gender, ethnicity, age and whether they have used any educational AR application before or not. All the questions in this section were self-designed.

**Section B** evaluates the variables of the research framework comprised of self-developed and adopted questions from multiple sources. Independent variable, human-human interaction measurement questions were formed based on the idea of peer-peer interaction and student-teacher interaction ( Table 3). How interactive technology promotes both types of interactivities in class was also examined by another study that targeted the usage of clickers in a classroom. ^3^

**Table 3.** Questionnaire of human-human interactivity factor.

Question	Pre-test	Post-test
1	This lecture facilitates interaction with peers	This application facilitates interaction with peers
2	This lecture facilitates interaction with teachers	This application facilitates interaction with teachers
3	This lecture provides opportunities to discuss with peers	This application provides opportunities to discuss with peers
4	This lecture provides opportunities to discuss with teacher	This application provides opportunities to discuss with teacher
5	This lecture allows exchange of information with peers	This application allows exchange of information with peers
6	This lecture allows exchange of information with teachers	This application allows exchange of information with teachers

For the second independent variable human-system interaction, the questions were adopted ^38^ and depicted in Table 4. The idea of human-system interaction is based on how satisfied the users are with using a particular system. For this purpose, this study has adopted the measurement items used in terms of suitability to the task or how efficient it is in performing the intended tasks, controllability, suitability for learning the usage of the application, and how well the system is for self-descriptiveness. ^38^

**Table 4.** Questionnaire of human-system interactivity factor.

Question	Adopted paper	This paper
7	Easy to use	*It is easy to use*
8	Offers functions to solve tasks efficiently	*It gives opportunity to understand the lesson more easily* through AR object
9	Provides a good overview over its functionality	*It provides a good overview over its functionality*
10	Offers an opportunity to stop the task and continue at the same point later on	*It offers an opportunity to stop the task and continue at the same point later on*
11	Easy to learn without somebody help or manual	*It is easy to learn without somebody’s help or manual*
12	The familiarization with gestures and manipulating virtual objects has been easy	*I find the augmented object realistic*
13	The three-dimensional virtual figures are clear and do not present definition difficulties	*The manipulation of augmented 3D solid was smooth*
14	Utilizing materials (design notebook) and augmented reality technology has been easy and intuitive	*It is easy to have 360 degree view by rotating the solid via finger touch*

The learning experience variable was evaluated based on the adopted questionnaire from ^31^ shown in Table 5. In their study, they measured learning motivation as part of AR learning experience. Motivation has been measured by a developed model of attention, relevance, confidence, and satisfaction. ^39^

**Table 5.** Questionnaire of learning experience factor.

No	Adopted paper	Current study
15	The quality of the augmented reality material helped to hold my attention	*The quality of the augmented reality material helped to hold my attention*
16	The variety of audio visual material helped keep my attention on the lesson	*The variety of audio visual material helped to keep my attention on the lesson*
17	The content of this material is relevant to my interests	*The AR content of this material was relevant to my lesson*
18	The content of this material is relevant to my interests	*The audio visual material in this lesson is related to my lesson*
19	As I worked on this lesson, I was confident that I could learn the content	*The AR and audio visual contents in IARA enabled me to learn independently*
20	After working on this lesson for a while, I was confident that I would be able to pass a test on it	*After working on this lesson for a while, I was confident that I would be able to pass a test on it*
21	It was a pleasure to work on such a well-designed lesson	*I felt good to interact with AR object*
22	It was a pleasure to work on such a well-designed lesson	*I enjoyed the audio visual content so much that I would like to know more about this topic*
23	It was a pleasure to work on such a well-designed lesson	*I felt good after sharing information through discussion platform*

The self-developed questionnaire listed in Table 6. All the questions for the aforementioned variables were measured through 5-point Likert scale.

**Table 6.** Self-developed questionnaire of learning experience factor.

No	Self-developed
24	*Video of object formulation through revolution of solid has deepen my understanding*
25	*AR object has facilitated in visualizing the solid better*
26	*I am satisfied with the content and function of this application*

**Section C** was developed by a calculus subject expert in evaluating learning performance via a quiz. The quiz included five True/False and one formative questions on solid of revolution chapter. The quiz was used in pre- and post-test evaluation for measuring learning performance factor. To avoid question biases, the same questions were used. Order of true-false questions were changed to make sure students did not just follow the similar pattern of answer from the pre-test.

### Data analysis techniques

This study conducted two types of analysis through Smart PLS 3.0 and SPSS 22 ( IBM SPSS Statistics, RRID: SCR_019096) for conducting structural model assessment and pre-and post-test comparison of variables respectively. R is an open-source alternative software (R Project for Statistical Computing, RRID: SCR_001905) for Smart PLS.

## Results

### Descriptive statistics

**Demographic profile**

This study collected 55 valid responses from the selected sample of 59 students from a Malaysian private university ( Table 7). Out of 55 respondents, 46 were male (83.6%) while 9 were female constituting 16.4% of total respondents. The result is in line with other research findings as the gender ratio significantly skews towards males in the technology field. ^40^^,^^41^ Among the three main ethnicity groups 43 students were Chinese (78.2%), followed by six Malay and six Indians constituting 10.9% each. As the sample was from pre-university students, the age range was from 18-22. The majority of students were 19 years old (60%) where 18 and 22 were the smallest age groups, constituting 3.6 % each.

**Table 7.** Demographic information.

Criterion		Frequency	Percentage
**Gender**	Male	46	83.6
	Female	9	16.4
**Race**	Malay	6	10.9
	Indian	6	10.9
	Chinese	43	78.2
**Age (years)**	18	2	3.6
	19	33	60.0
	20	12	21.8
	21	6	10.9
	22	2	3.6

Students’ experience of using similar types of applications in learning provides an indication of whether users are experienced in using this type of application or not. It is found that the students scored the same grades in this pre-assessment, indicating that they had the same initial level of understanding in the studied area before using the system.

### Inferential statistics

The study adopted a PLS_SEM approach to maximize the variance of the defined framework’s endogenous construct. ^42^ Structural model analysis was used to test the hypotheses along with its predictive accuracy and effect size. All the constructs were measured with five or more indicators. As the model is reflective, the constructs are reflective too. “Internal consistency reliability”, “convergent validity” and “Discriminant Validity” are the three criteria used to assess the constructs. ^37^

**Internal consistent reliability**

To evaluate the same source biasedness coefficient variation, Internal consistency reliability is used to investigate the reliability of indicators that measure a latent variable. From Table 8, each construct satisfied the criteria of composite reliability (CR) of ≥ 0.700. ^43^ As such it can be concluded that the constructs met the internal consistency reliability criteria. All the self-developed questions under human-human interaction variables satisfied the reliability as the loading is >0.600. ^36^ For the three self-developed questions (LE 10, LE11, and LE12) under learning experience variable two of the questions LE11, LE12 also satisfy this criterion.

**Table 8.** Convergent validity and composite reliability.

Construct	Item	Loading	CR	AVE
**Human-Human Interaction**	HHI1	0.678	0.886	0.564
	HHI2	0.783		
	HHI3	0.771		
	HHI4	0.796		
	HHI5	0.724		
	HHI6	0.747		
**Human-System Interaction**	HSI1	0.665	0.886	0.526
	HSI2	0.728		
	HSI3	0.800		
	HSI4	0.801		
	HSI5	0.683		
	HSI6	0.691		
	HSI7	0.697		
**Learning Experience**	LE11	0.740	0.891	0.505
	LE12	0.690		
	LE2	0.735		
	LE5	0.649		
	LE6	0.693		
	LE7	0.732		
	LE8	0.673		
	LE9	0.766		

**Convergent validity**

Convergent validity is measured by the indicator of Average Variance Extracted (AVE) and factor loading. ^43^ AVE indicates what percentage of the variance of a construct is defined by a marker. ^43^ Acceptable value of AVE for each construct must be ≥ 0.500. From the AVE value depicted in Table 8, all constructs satisfied the convergent validity criteria. Factor loading of each indicator ≥ 0.6 is acceptable. ^44^

**Discriminant validity**

Discriminant validity is measured by the Fornell and Larcker Criterion (FLC), cross-loading comparison and HTMT technique. ^36^ FLC indicates that the square roots of AVEs for the reflective constructs must be larger than the correlation for all other constructs diagonally. Table 9 shows that all constructs satisfied FLC criteria, where the square roots of AVEs for the reflective constructs of HHI (0.751), HSI (0.726) and LE (0.711) satisfied this criteria.

**Table 9.** Discriminant validity-fornell and larcker criterion.

	Human-Human Interaction	Human-System Interaction	Learning Experience
**Human-Human Interaction**	**0.751**		
**Human-System Interaction**	0.565	**0.726**	
**Learning Experience**	0.714	0.616	**0.711**

From Table 10 of cross loading, all indicators load high on their own constructs compared to others. This confirms that the constructs are distinct from each other indicating discriminant validity as a result. All the items including the self-developed questions satisfy this validity criteria.

**Table 10.** Convergent validity cross loading.

	Human-Human Interaction	Human-System Interaction	Learning Experience
**HHI1**	0.678	0.412	0.422
**HHI2**	0.783	0.466	0.552
**HHI3**	0.771	0.437	0.504
**HHI4**	0.796	0.447	0.636
**HHI5**	0.724	0.474	0.492
**HHI6**	0.747	0.326	0.574
**HSI1**	0.259	0.665	0.214
**HSI2**	0.494	0.728	0.444
**HSI3**	0.432	0.800	0.498
**HSI4**	0.377	0.801	0.330
**HSI5**	0.343	0.683	0.438
**HSI6**	0.484	0.691	0.547
**HSI7**	0.376	0.697	0.475
**LE11**	0.431	0.501	0.74
**LE12**	0.423	0.434	0.69
**LE2**	0.608	0.379	0.735
**LE5**	0.514	0.465	0.649
**LE6**	0.549	0.476	0.693
**LE7**	0.463	0.575	0.732
**LE8**	0.414	0.26	0.673
**LE9**	0.605	0.375	0.766

HTMT mechanism of assessing discriminant validity requires the value of HTMT to be lower than 0.850 for stringent criterion and 0.900 for conservative criterion. ^36^ From Table 11, all the constructs satisfy the above criteria confirming discriminant validity thereof.

**Table 11.** Discriminant validity-HTMT.

	Human-Human Interaction	Human-System Interaction	Learning Experience
**Human-Human Interaction**			
**Human-System Interaction**	**0.646**		
**Learning Experience**	**0.817**	**0.672**	

From all the criteria of confirmatory factorial analysis, the research model is adequately fitting to be accepted. As such this designated measurement model with specified latent variables has been analyzed with SEM criteria.

**Structural Model Assessment**

Path analysis was performed to find the hypothesized relationship. The results for collinearity assessment and hypothesis are shown in Table 12.

**Table 12.** Collinearity assessment and hypothesis test.

Latent variables	VIF	Beta ß	t-value	p-value	*f ^2^*
Human-Human Interaction -> Learning Experience ***	1.470	0.537	4.629	0.000	0.463
Human-System Interaction -> Learning Experience **	1.470	0.313	2.588	0.005	0.157

Both latent variables Human-Human Interaction (HHI) and Human-System Interaction (HSI) have positive effects on Learning Experience (LE). The variance inflation factor (VIF) results in Table 12 show that the lateral multicollinearity meets the criteria of being above the threshold of 0.2 and below the threshold of 5, implying collinearity was not an issue in the structural model. ^43^

A study by ^43^ suggested that for a one-tailed test “ *t-values”* for a significant level of five per cent (α = 0.05) are required to be greater than 1.645. The result indicated that both exogenous constructs HHI and HSI have a “ *t-value*” of >1.645 for a significant level of five per cent (α = 0.05). From the results, both latent variables have a positive relationship with LE, HHI being the stronger predictor than HSI.

**Predictive accuracy, effect size and relevance**

“Predictive accuracy” is evaluated through the “coefficient of determination, R ^2^”. R ^2^ values imply the predictive power of exogenous constructs over endogenous ones. From the analysis construct LE’s R ^2^ value is found as 0.576 which means exogenous constructs substantially explain 57.6% of LE’s variance as predictive power can be considered as substantial if it is greater than 0.260. ^45^ Although ^43^ has stated R ^2^ value less than 0.670 is moderate.

To evaluate the effect size of exogenous constructs Cohen’s *f* ^2^ value was obtained from the model analysis. ^45^ stated that the *f* ^2^ value of 0.350 is considered a substantial effect whereas 0.150 is considered moderate. From Table 12 it can be seen that the HHI construct has a substantial effect on LE (0.463) and HSI has a moderate effect (0.157).

In addition, the Q ^2^ value of endogenous construct LE was found at 0.269 indicating moderate “predictive relevance”. ^43^ Besides, as the Q ^2^ value is larger than 0, it can be concluded that HHI and HIS exogenous constructs have “predictive relevance” for the endogenous construct LE. ^46^

**Pre-test post-test**

For understanding the significance of pre-and post-test performance of student paired sample t-test was carried out and the result is shown in Table 13. From Table 13 it can be concluded that there is a significant relationship between the results of the pre-and post-tests as the P<0.050. ^32^ The post-test mean implies that students’ performance results in the post-test are higher than the pre-test one signifying a positive improvement in the performance of learning.

**Table 13.** Pre and post test analysis of performance.

Paired samples test									
		Paired differences					t	df	Sig. (2-tailed)
		Mean	Std. deviation	Std. error mean	95% confidence interval of the difference				
					Lower	Upper			
Pair 1	Pre-test Post-test	−1.436	1.437	.194	−1.825	−1.048	−7.412	54.000	0.000

## Discussion

The study aims to develop an augmented reality application that promotes interaction and spatial visualization for learning calculus and evaluates the effect of the interactive learning system on the performance of learning. Human-human interaction is evaluated as part of the chat function of application and human-system interaction is analyzed as part of augmented reality implementation through mobile application.

The first hypothesis was accepted as human-system interactivity positively affected the learning experience factor. As this study developed an augmented reality application for learning calculus, here human-system interaction is related to the interactivity promoted by the augmented reality application. Studies implementing augmented reality in improving learning experience founds that the didactic experience of this technology make students more engaged and hence providing an enriched learning experience. ^12^^,^^14^^,^^16^^,^^21^^,^^27^ This study has also found that among the human-system interaction all students univocally cancelled out disagreement that it provided them with real world 3D object feelings. The study using similar questionnaire have also found that it has significant impact on the learning experience in general and motivation in particular. ^39^ So, the result of this study is aligned with the prior relevant studies.

The second hypothesis result found that human-human interaction significantly influences learning experience. The implication from this hypothesis acceptance indicate that learning experience is shaped by human-human interaction which can comes from student-teacher or student-student. In the study design both student-teacher and student-student interaction facilities were provided via application chat function. So, it can be said that both type of human-human interactions are associated positively with learning experience. This result is in line with the findings where both types of interaction led to learning satisfaction. ^47^ Another study found that human-human interactivity increases learning engagement. ^1^^–^^3^ In addition to that another study has also claimed that learning confidence as part of learning experience has also increased through interaction with peers and teachers. ^1^ From the findings of these research, it can be concluded that the result of human-human interaction affecting learning experience is in line with existing research.

The third hypothesis of the study was tested by using paired sample *“t-test”* where learning performance’ means were compared from ‘pre-test’ to ‘post-test’ scenario. The result shows that there is significant relation between the score and “post-test” mean is higher than the ‘pre-test’ one. Other studies have also found that learning experience as result of human-system and human-human interactivity increase learning performance. ^3^^,^^15^^,^^47^ So, it can be claimed that in terms of hypothesis acceptance, this study is in line with existing literature.

## Conclusions

The role of interactivity in the learning experience has been established by many studies before, but implementing both human-human and human-system interactivities in an augmented reality application was overdue. This research had done exactly that and analyzed the effectiveness of the research framework by using PLS. From the results, it can be concluded that the model was fit to analyze the research framework with substantial predictive accuracy and a moderate effect size of the exogenous variable with a moderate relevance on endogenous variable LE. All three hypotheses are confirmed as P values for all three are at a satisfactory level. These results imply that human-human and human-system interactions positively affect learning experience and performance of learning as a result of an enhanced learning experience.

## Ethical considerations and consent

All the procedures performed in this study involving human participants were in adherence to the ethical policies of the University as approved by the Technology Transfer Office of Multimedia University under ethical approval number: EA0702021.

Written consent was also obtained from all individual participants involved in the study. Personal data from individuals was promised to be kept confidential and strictly restricted for use in this study only.

## Data Availability

Zenodo: IARA LP Dataset. https://doi.org/10.5281/zenodo.5744960 ^48^ This project contains the following underlying data: Dataset IARA LP 01122021.xlsx (The file contains two sheets. The first one contains the indicators of three variables; Learning Experience, Human-Human Interaction and Human-System Interaction which were used for framework analysis. The second sheet includes the results of students’ performance before and after using the learning system as pre-test and post-test). Data are available under the terms of the Creative Commons Attribution 4.0 International license (CC-BY 4.0).
