# Supplementary material for: Interactive Learning System for Learning Calculus
Source: F1000Res. 2024 Mar 11;11:307. Originally published 2022 Mar 14. [Version 2] doi: 10.12688/f1000research.73595.2 (PMC11101922; doi:10.12688/f1000research.73595.2)
Supplement: Peer review history

# Reviewer report: Reviewer response for version 2

**DOI:** 10.5256/f1000research.162235.r261044

**Published:** 2024-05-17

**Authors:** Lukman Hakim Muhaimin

**Affiliations:** 1 Universitas Pendidikan Indonesia, Bandung, Indonesia

The study design, methodology, data interpretation, and conclusion sections are appropriate and effectively convey the research intent. I understand the researcher focuses here on discussing interactive media, with implicit reference to AR in the title. However, I suggest adding a discussion on the theories related to AR and linking them with interactive learning within the theoretical framework section. Furthermore, I recommend including a brief depiction of the interactive media (such as displaying images) to enhance the readers' visualization.

 Lastly, I'd like to inquire: If these media were removed from the process of understanding calculus concepts, would students still be able to grasp those concepts? I hope the media developed by the researcher serves as an extended cognition, not merely a teaching aid that, if removed, would leave students unable to understand mathematical concepts.

Is the work clearly and accurately presented and does it cite the current literature?

Partly

If applicable, is the statistical analysis and its interpretation appropriate?

I cannot comment. A qualified statistician is required.

Are all the source data underlying the results available to ensure full reproducibility?

Yes

Is the study design appropriate and is the work technically sound?

Yes

Are the conclusions drawn adequately supported by the results?

Yes

Are sufficient details of methods and analysis provided to allow replication by others?

Yes

Reviewer Expertise:

mathematics education, ICT

I confirm that I have read this submission and believe that I have an appropriate level of expertise to confirm that it is of an acceptable scientific standard, however I have significant reservations, as outlined above.

# Reviewer report: Reviewer response for version 2

**DOI:** 10.5256/f1000research.162235.r254520

**Published:** 2024-04-11

**Authors:** Yong Wee Sek

**Affiliations:** 1 Fakulti Teknologi Maklumat dan Komunikasi, Universiti Teknikal Malaysia, Melaka, Malaysia

The authors have addressed all comments and provide justifications for each section of the journal.  However, the discussion section can be further improved. The quality of this paper can be further enhanced by providing explanations of the results in the discussion section. for example, for the third hypothesis, explain why this phenomena happen.

Is the work clearly and accurately presented and does it cite the current literature?

Partly

If applicable, is the statistical analysis and its interpretation appropriate?

Partly

Are all the source data underlying the results available to ensure full reproducibility?

Partly

Is the study design appropriate and is the work technically sound?

Partly

Are the conclusions drawn adequately supported by the results?

Partly

Are sufficient details of methods and analysis provided to allow replication by others?

Partly

Reviewer Expertise:

My research interest is in educational technologies, technology acceptance and information system

I confirm that I have read this submission and believe that I have an appropriate level of expertise to confirm that it is of an acceptable scientific standard.

# Reviewer report: Reviewer response for version 1

**DOI:** 10.5256/f1000research.77256.r127478

**Published:** 2022-04-12

**Authors:** Affandy Affandy

**Affiliations:** 1 Faculty of Computer Science, Dian Nuswantoro University, Semarang, Indonesia

This research explores the benefits of human interaction in interactive learning systems with visualization through augmented reality. The subject exposed is calculus on the topic "Revolution of Solids". The research compares the impact of human-system interaction support with human-human interaction support on student learning experiences.

 In this paper, the author presents information that is quite valuable, however, several important things need to be detailed to get a more comprehensive point of view.

**Introduction:**

 In this section, explanations related to the following things need to be explained to construct a more complete research motivation:

1. What problems are still encountered in the application of learning media based on 3D visualization with AR technology?
2. What systems currently exist to support learning calculus using AR and what are their weaknesses?
3. What are the existing systems that support visualization and interaction and what are the problems with these systems?

**Literature study:**

 The paper needs to include an explanation of the following points to add clarity to the problem and state-of-the-art research on learning using visualization:

1. Utilization of AR technology in calculus learning.
2. Various interaction models exist in learning applications and their advantages and disadvantages.
3. Why visualization systems must be supported by human-human interaction in learning applications?
4. What prevents current AR applications from providing student-student and teacher-student interaction?

**Methodology:**

 To ensure the homogeneity of the sample, it was mentioned in the paper that none of the students had ever used AR applications before. However, the student's initial level of understanding in the area studied before they used the provided AR applications is unknown. Different starting points have the potential to provide different endpoints as well.

**Discussion:**

 In the discussion section, the author should not only emphasize the findings obtained from the statistical approach that has been carried out. A more in-depth study to find out what aspects make human-human interaction give better student performance results than human-system interaction needs to be presented with comparisons from previous studies.

 Using previous studies as a reference in the discussion/comparison of research results will provide clarity on the contribution of research in this field of study.

Is the work clearly and accurately presented and does it cite the current literature?

Partly

If applicable, is the statistical analysis and its interpretation appropriate?

I cannot comment. A qualified statistician is required.

Are all the source data underlying the results available to ensure full reproducibility?

Partly

Is the study design appropriate and is the work technically sound?

Partly

Are the conclusions drawn adequately supported by the results?

Partly

Are sufficient details of methods and analysis provided to allow replication by others?

Partly

Reviewer Expertise:

My research interest is in software engineering education, software visualization, and project management.

I confirm that I have read this submission and believe that I have an appropriate level of expertise to confirm that it is of an acceptable scientific standard, however I have significant reservations, as outlined above.

# Response

**Published:** 2024-02-02

**Authors:** Sook Ling Lew

**Affiliations:** Multimedia University, Malaysia

Comment 1: This research explores the benefits of human interaction in interactive learning systems with visualization through augmented reality. The subject exposed is calculus on the topic "Revolution of Solids". The research compares the impact of human-system interaction support with human-human interaction support on student learning experiences.

 In this paper, the author presents information that is quite valuable, however, several important things need to be detailed to get a more comprehensive point of view.

 Response 1: We have revised the manuscript carefully based on all the comments and issues raised.

 Comment 2: Introduction:

 In this section, explanations related to the following things need to be explained to construct a more complete research motivation:

 What problems are still encountered in the application of learning media based on 3D visualization with AR technology?

 Response 2: The problems are included as follows:

 (1)    Lacking Human-Human interactivity.

 Introduction – Paragraph 1:

 Although there are some studies that have addressed the issues of student interaction with the system there is still a lack of teacher-student or student-student interaction in interactive learning systems [1, 2, 3]. Many systems have promoted a single type of interactivity [4, 5, 6, 7, 8, 9, 10].

 Literature Review – Interactivity - Paragraph 1:

 Past studies in Table 1 shows promoted interactivities by different studies. These studies focused primarily on human-system interactivity while a few provided human-human interactivity.

 Literature Review – Interactivity - Paragraph 2:

 Past studies in Table 2 depicts that most systems solely focused on interactivity through AR and not implementing any human-human interactivity.

 (2)    Lacking Spatial Visualization

 Introduction – Paragraph 2:

 Application of integration is one of the chapters in Calculus that requires spatial visualization and the 2D medium of the traditional classroom does not provide a proper solution [11, 12, 13].

 Introduction – Paragraph 3:

 After consulting with the subject teacher on what the student struggle most in learning calculus, it was suggested that students face difficulties in understanding solid of revolution through traditional classroom method as it requires 3D visualization.

 Comment 3: What systems currently exist to support learning calculus using AR and what are their weaknesses?

 Response 3: Literature Review – Interactivity - Paragraph 1:

 The existing interactive methods proposed to overcome these problems are included as:

 AR based interactive book [5] is a tool used in asynchronous learning. Past studies in Table 1 shows promoted interactivities by different studies. These studies focused primarily on human-system interactivity while a few provided human-human interactivity.

 Introduction – Paragraph 4:

 Advantages of the existing methods are included as:

 Interactive technology has made learning more personalized and kept students engaged through various applications [23]. This paper also suggested that interactive technology can make learning more active, and intensive for students where students can communicate easily with each-others and also with the teacher [23].

 Disadvantages of the existing methods are included as:

 Recently implementation of Augmented Reality (AR) in pedagogy has been adopted by many researchers where the emphasis has mainly been kept on 3D visualization. Many have implemented AR in geometry for school students [14, 19, 24] while some other implemented it for calculus [12, 13]. In both cases the authors have focused on the immersive learning experience through AR but no human-human interaction function was provided.

 Comment 4: What are the existing systems that support visualization and interaction and what are the problems with these systems?

 Response 4: Introduction – Paragraph 3:

 Although researchers have implemented AR to assist in visualization, they are mainly focused on human-system interaction, either through haptic or non-haptic interaction [14, 15, 16, 17]. Besides, agility is another issue that some systems could not addresses as they are desktop-based systems [17, 18, 19]. Under the theory of learner constructing their knowledge Lev Vygotsky theorized that learning occurs through personalization and socialization [20]. So it is crucial to provide the function to interact among the learners.

 Comment 5: Literature study:

 The paper needs to include an explanation of the following points to add clarity to the problem and state-of-the-art research on learning using visualization:

 Utilization of AR technology in calculus learning.

 Response 5: Advantages of the existing methods are included as:

 Interactive technology has made learning more personalized and kept students engaged through various applications [23]. This paper also suggested that interactive technology can make learning more active, and intensive for students where students can communicate easily with each-others and also with the teacher [23].

 Disadvantages of the existing methods are included as:

 Recently implementation of Augmented Reality (AR) in pedagogy has been adopted by many researchers where the emphasis has mainly been kept on 3D visualization. Many have implemented AR in geometry for school students [14, 19, 24] while some other implemented it for calculus [12, 13]. In both cases the authors have focused on the immersive learning experience through AR but no human-human interaction function was provided.

 Comment 6: Various interaction models exist in learning applications and their advantages and disadvantages.

 Response 6: Under the theory of learner constructing their knowledge Lev Vygotsky theorized that learning occurs through personalization and socialization [20] . So it is crucial to provide the function to interact among the learners. Unfortunately, majority of the AR learning application only focused on providing content visualization resulting in not a holistic application in learning that promote both type of interaction [14, 15, 21, 22].

 Comment 7: Why visualization systems must be supported by human-human interaction in learning applications?

 Response 7: Under the theory of learner constructing their knowledge Lev Vygotsky theorized that learning occurs through personalization and socialization [20] . So it is crucial to provide the function to interact among the learners.

 Comment 8: What prevents current AR applications from providing student-student and teacher-student interaction?

 Response 8: The current AR applications emphasized on immersive learning experience through AR that lacking in providing student-student and teacher-student interaction. The information is included as follows:

 Recently implementation of Augmented Reality (AR) in pedagogy has been adopted by many researchers where the emphasis has mainly been kept on 3D visualization. Many have implemented AR in geometry for school students [14, 19, 24] while some other implemented it for calculus [12, 13]. In both cases the authors have focused on the immersive learning experience through AR but no human-human interaction function was provided.

 Comment 9: Methodology:

 To ensure the homogeneity of the sample, it was mentioned in the paper that none of the students had ever used AR applications before. However, the student's initial level of understanding in the area studied before they used the provided AR applications is unknown. Different starting points have the potential to provide different endpoints as well.

 Response 9: Homogeneity of the sample is included as follows:

 Methods-Research Design and Participants

 This study conducted a pre-assessment evaluation of the respondents' knowledge, skills, or understanding in the AR area related to the system before they started using it [32].

 Descriptive Statistics-Demographic Profile

 It is found that the students scored the same grades in this pre-assessment, indicating that they had the same initial level of understanding in the studied area before using the system.

 Comment 10: Discussion:

 In the discussion section, the author should not only emphasize the findings obtained from the statistical approach that has been carried out. A more in-depth study to find out what aspects make human-human interaction give better student performance results than human-system interaction needs to be presented with comparisons from previous studies.

 Response 10: The discussion was rewritten with comparisons from previous studies as follows:

 The study aims to develop an augmented reality application that promotes interaction and spatial visualization for learning calculus and evaluates the effect of the interactive learning system on the performance of learning. Human-human interaction is evaluated as part of the chat function of application and human-system interaction is analysed as part of augmented reality implementation through mobile application.

 The first hypothesis was accepted as human-system interactivity positively affected the learning experience factor. As this study developed an augmented reality application for learning calculus, here human-system interaction is related to the interactivity promoted by the augmented reality application. Studies implementing augmented reality in improving learning experience founds that the didactic experience of this technology make students more engaged and hence providing an enriched learning experience [12, 14, 16, 21, 27]. This study has also found that among the human-system interaction all students univocally cancelled out disagreement that it provided them with real world 3D object feelings. The study using similar questionnaire have also found that it has significant impact on the learning experience in general and motivation in particular [39]. So, the result of this study is aligned with the prior relevant studies.

 The second hypothesis result found that human-human interaction significantly influences learning experience. The implication from this hypothesis acceptance indicate that learning experience is shaped by human-human interaction which can comes from student-teacher or student-student. In the study design both student-teacher and student-student interaction facilities were provided via application chat function. So, it can be said that both type of human-human interactions are associated positively with learning experience. This result is in line with the findings where both types of interaction led to learning satisfaction [47]. Another study found that human-human interactivity increases learning engagement [1, 2, 3]. In addition to that another study has also claimed that learning confidence as part of learning experience has also increased through interaction with peers and teachers [1]. From the findings of these research, it can be concluded that the result of human-human interaction affecting learning experience is in line with existing research.

 The third hypothesis of the study was tested by using paired sample “t-test” where learning performance means were compared from ‘pre-test’ to ‘post-test scenario. The result shows that there is significant relation between the score and “post-test” mean is higher than the ‘pre-test’ one. Other studies have also found that learning experience as result of human-system and human-human interactivity increase learning performance [3, 15, 47]. So, it can be claimed that in terms of hypothesis acceptance, this study is in line with existing literature.

 Comment 11: Using previous studies as a reference in the discussion/comparison of research results will provide clarity on the contribution of research in this field of study.

 Response 11: The discussion was rewritten with comparisons from previous studies as above in Response 10.

# Reviewer report: Reviewer response for version 1

**DOI:** 10.5256/f1000research.77256.r127477

**Published:** 2022-03-28

**Authors:** Yong Wee Sek

**Affiliations:** 1 Fakulti Teknologi Maklumat dan Komunikasi, Universiti Teknikal Malaysia, Melaka, Malaysia

In this paper, the authors introduced the interactive learning system for learning Calculus. There is a great deal of good work in this paper. However, it does require a significant overall revision to make the best of this and present the work in a clear and well-structured way, particularly on the literature review, methodology, research design and procedures used in this study, measurement instruments, and data analysis techniques in order to achieve the research objectives. Additionally, the discussion section is not exhaustive enough to provide good attempt to explain many of the study results. In fact, the discussion is not aligned with the research objectives.

**Abstract**

 Background – the problems of the interactive system are not clear. The authors should point out the existing problems of the interactive system in learning mathematics, particularly in learning Calculus.

 Please include the main objective(s) or research question(s) of this study in this section. The research objectives/research questions are not clear.

**Introduction**

 This section should include the following information:

- What are the existing problems in learning Calculus?
- Why is “Revolution of Solids” proposed in this research?
- Why is an interactive approach proposed to overcome these problems?
- What are the existing interactive methods that have been proposed to solve Mathematics problem, in particular Calculus (Revolution of Solids)?
- What are the weaknesses of existing interactive methods in solving mathematics (Calculus - Revolution of Solids) problems?
- How are the proposed interactive methods (augmented reality and chat functions) able to solve the existing problem in learning Revolution of Solids?

Please include the main objective(s)/research questions of this study in this section.

 Include a brief description of the methodology and system used in this study.

**Literature review/Background study**

 In this section, discussion on the following should be included:

- The major problems in learning calculus (visualization)
- How are the interactive activities able to solve the existing problem in learning calculus?
- What are the existing interactive methods proposed to overcome these problems? Should discuss the advantages and disadvantages of the existing methods.
- What are the major issues of the existing interactive methods?
- Why are AR and chat functions introduced in learning calculus?

 

**Methodology**

 The author may consider sub-dividing this section as suggested below:

1. Study design and procedures
2. The design and development of the interactive methods
3. Participants
4. Measurement scales
5. Data analysis techniques

Study design and procedures – you should discuss the choice of quantitative research method in this research, what is the quasi-experimental design? Why is quasi-experimental design used in this research? What are the procedures used to conduct this research?

 A discussion on the design and development of the interactive methods should be presented here.

 Participants – this study uses quasi-experimental design, the participants should be assigned into control and experimental group. This information is not available. How did you assign participants into these groups? How did you select the participants for this study?

 For measurement items, what are the scales used in this study? Please list the sources. For the self-developed items, reliability and validity tests should be conducted and reported.

 What is the structural equation modelling (SEM)? Why is SEM used in this research? How did you conduct the data analysis? You should also discuss the data analysis for longitudinal study using SEM.

**Results**

 As the measurement items were adopted from many different sources, instrument reliability and validity tests using Exploratory Factor Analysis (EFA) and Confirmatory Factor Analysis (CFA) should be conducted and reported.

 For self-developed items, procedures to establish instrument reliability and validity should be presented.

 The result section needs to do a major revision with appropriate analyses. Please include relevant Table(s) with appropriate details such as paired t test results, significant value, etc. for each of the test conducted.

**Discussions**

 The author(s) should discuss/relate/compare the findings with prior relevant studies. Please also explain how the results in this study relate to previous findings, whether in support, contradiction, or simply as added data. The discussion should be aligned with the research objectives.

**Additional comments:**

- Page 5, figure 1. Research design - Causal not casual relationship
- The development of research hypotheses is missing.
- Did not justify the adopted questionnaire.
- Did not specify the questions for the quiz. Are the same or different sets of questions used for pre and post? How many questions?
- Did not mention if measurement model and structural model were assessed based on data collected in the first phase or the third phase?
- Ensure consistency in reporting the number of decimals throughout the manuscript (including in Tables).
- Please provide information about the ethical applications
- Please refer to the article entitled "Designing and evaluating a high interactive augmented reality system for programming learning" ^1^. This paper will provide some ideas on how to conduct quasi-experimental design, research procedures, and how the discussion was presented. 
- For the article entitled "Primary school students' perceptions of scaffolding in digital game-based learning in mathematics" ^2^, This paper can give some information about the process of the research experiment, ethical considerations, and how the discussion was presented.

Is the work clearly and accurately presented and does it cite the current literature?

Partly

If applicable, is the statistical analysis and its interpretation appropriate?

Partly

Are all the source data underlying the results available to ensure full reproducibility?

Partly

Is the study design appropriate and is the work technically sound?

Partly

Are the conclusions drawn adequately supported by the results?

Partly

Are sufficient details of methods and analysis provided to allow replication by others?

Partly

Reviewer Expertise:

My research interest is in educational technologies, technology acceptance and information system

I confirm that I have read this submission and believe that I have an appropriate level of expertise to confirm that it is of an acceptable scientific standard, however I have significant reservations, as outlined above.

## References

[1] : Designing and evaluating a high interactive augmented reality system for programming learning. *Computers in Human Behavior* .2022;132: 10.1016/j.chb.2022.107245 10.1016/j.chb.2022.107245

[2] : Primary school students' perceptions of scaffolding in digital game-based learning in mathematics. *Learning, Culture and Social Interaction* .2021;28: 10.1016/j.lcsi.2020.100457 10.1016/j.lcsi.2020.100457

# Response

**Published:** 2024-02-02

**Authors:** Sook Ling Lew

**Affiliations:** Multimedia University, Malaysia

Comment 1: In this paper, the authors introduced the interactive learning system for learning Calculus. There is a great deal of good work in this paper. However, it does require a significant overall revision to make the best of this and present the work in a clear and well-structured way, particularly on the literature review, the methodology, the research design and procedures used in this study, measurement instruments and data analysis techniques to achieve the research objectives. Additionally, the discussion section is not exhaustive to provide good attempt to explain many of the study results. In fact, the discussion is not aligned with the research objectives.

 Response 1: We have revised the manuscript carefully based on all the comments and issues raised.

 Comment 2: Abstract

 Background – the problems of the interactive system are not clear. The authors should point out the existing problems of the interactive system in learning mathematics, particularly in learning Calculus.

 Please include the main objective(s) or research question(s) of this study in this section. The research objectives/research questions are not clear.

 Response 2: Existing problems of the interactive system in learning mathematics, particularly in learning Calculus are added as follows:

 However, calculus learning augmented reality application has limitation in promoting a human collaboration in learning. 

 The main objective is included in Purpose and Introduction:

 This research develops an augmented reality application that promotes interaction and spatial visualization for learning calculus and evaluates the effect of the interactive learning system on the performance of learning.

 Comment 3: Introduction

 This section should include the following information:

 Response 3: The existing problems in learning Calculus are included Introduction – Paragraph 1, 2, 3, Literature Review – Interactivity - Paragraph 1 & Paragraph 2; as follows:

 Comment 4: What are the existing problems in learning Calculus?

 Response 4:

 1.       Lacking Human-Human interactivity.

 Although there are some studies that have addressed the issues of student interaction with the system there is still a lack of teacher-student or student-student interaction in interactive learning systems [1, 2, 3]. Many systems have promoted a single type of interactivity [4, 5, 6, 7, 8, 9, 10]. Past studies in Table 1 shows promoted interactivities by different studies. These studies focused primarily on human-system interactivity while a few provided human-human interactivity. Past studies in Table 2 depicts that most systems solely focused on interactivity through AR and not implementing any human-human interactivity.

 2.       Lacking Spatial Visualization

 Application of integration is one of the chapters in Calculus that requires spatial visualization and the 2D medium of the traditional classroom does not provide a proper solution [11, 12, 13]. After consulting with the subject teacher on what the student struggle most in learning calculus, it was suggested that students face difficulties in understanding solid of revolution through traditional classroom method as it requires 3D visualization.

 Comment 5: Why “Revolution of Solids” is proposed in this research?

 Response 5: The reasons of “Revolution of Solids” are included as follows:

 In understanding revolution of solids around an axis, visualization of the solid in-question is very important. Spatially related 3D images cannot be drawn on a 2D board or projected over a computer. These hinder students in visualizing the solid and result in difficulties in conceptualizing the concept.

 After consulting with the subject teacher on what the student struggle most in learning calculus, it was suggested that students face difficulties in understanding solid of revolution through traditional classroom method as it requires 3D visualization.

 Comment 6: Why interactive approach is proposed to overcome these problems?

 Response 6: The reasons of interactive approach are included as follows:

 Although researchers have implemented AR to assist in visualization, they are mainly focused on human-system interaction, either through haptic or non-haptic interaction [14, 15, 16, 17]. Besides, agility is another issue that some systems could not addresses as they are desktop-based systems [17, 18, 19]. Under the theory of learner constructing their knowledge Lev Vygotsky theorized that learning occurs through personalization and socialization [20]. So it is crucial to provide the function to interact among the learners.

 Comment 7: What are the existing interactive methods have been proposed to solve Mathematics problem in particular Calculus (Revolution of Solids)?

 Response 7: The existing interactive methods are focused on providing content visualization and included in the Introduction as follows:

 Unfortunately, majority of the AR learning application only focused on providing content visualization resulting in not a holistic application in learning that promote both type of interaction [14, 15, 21, 22]. As such, developing an interactive AR application that facilitates both human-human and human-system interactivities and assists in conceptualizing and understanding math better is needed.

 Comment 8: What are the weaknesses of the existing interactive methods in solving mathematics (Calculus- Revolution of Solids) problems?

 Response 8: The weaknesses of the existing interactive methods are included in Introduction – Paragraph 4 as follows:

 Recently implementation of Augmented Reality (AR) in pedagogy has been adopted by many researchers where the emphasis has mainly been kept on 3D visualization. Many have implemented AR in geometry for school students [14, 19, 24] while some other implemented it for calculus [12, 13]. In both cases the authors have focused on the immersive learning experience through AR but no human-human interaction function was provided.

 Comment 9: How are the proposed interactive methods (augmented reality and chat functions) able to solve the existing problem in learning Revolution of Solids?

 Response 9: Introduction – Paragraph 3

 So, it is crucial to provide the function to interact among the learners. Unfortunately, majority of the AR learning application only focused on providing content visualization resulting in not a holistic application in learning that promote both type of interactions.

 The interactive methods (augmented reality and chat functions) are proposed as follows:

 1.       AR – to implement Spatial Visualization

 2. Chat – to improve Human-Human interactivity.

 Comment 10: Please include the main objective(s)/ research questions of this study in this section.

 Response 10: Main objectives are included as follows:

 This research aims to develop an interactive AR application for learning calculus that promotes human-system interaction via AR and human-human interaction through chat functions.

 Comment 11: Include a brief description of the methodology and system used in this study.

 Response 11: A brief description of the methodology and system used in this study are included as follows:

 Pre-test and post-test data from students learning calculus in the classroom using the AR interactive learning system were gathered using a questionnaire and a quiz to evaluate the impact of the developed AR system.

 Comment 12: Literature review/Background study

 In this section, discussion on the following should be included:

 Major problems in learning calculus (visualization)

 Response 12: Discussion on the major problems in learning calculus (visualization) are included as follows:

 1.       Lacking Human-Human interactivity.

 2.       Lacking Spatial Visualization

 Comment 13: How are the interactive activities able to solve the existing problem in learning calculus?

 Response 13: Under the theory of learner constructing their knowledge Lev Vygotsky theorized that learning occurs through personalization and socialization [20]. So, it is crucial to provide the function to interact among the learners. Unfortunately, majority of the AR learning application only focused on providing content visualization resulting in not a holistic application in learning that promote both type of interaction [14, 15, 21, 22].

 Comment 14: What are the existing interactive methods proposed to overcome these problems? Should discuss the advantages and disadvantages of the existing methods.

 Response 14: The existing interactive methods proposed to overcome these problems are included as:

 AR based interactive book [5] is a tool used in asynchronous learning. Past studies in Table 1 shows promoted interactivities by different studies. These studies focused primarily on human-system interactivity while a few provided human-human interactivity.

 Advantages of the existing methods are included as:

 Interactive technology has made learning more personalized and kept students engaged through various applications [23]. This paper also suggested that interactive technology can make learning more active, and intensive for students where students can communicate easily with each-others and also with the teacher [23].

 Disadvantages of the existing methods are included as:

 Recently implementation of Augmented Reality (AR) in pedagogy has been adopted by many researchers where the emphasis has mainly been kept on 3D visualization. Many have implemented AR in geometry for school students [14, 19, 24] while some other implemented it for calculus [12, 13]. In both cases the authors have focused on the immersive learning experience through AR but no human-human interaction function was provided.

 Comment 15: What are the major issues of the existing interactive methods?

 Response 15: Unfortunately, majority of the AR learning application only focused on providing content visualization resulting in not a holistic application in learning that promote both type of interaction [14, 15, 21, 22].

 Past studies in Table 2 depicts that most systems solely focused on interactivity through AR and not implementing any human-human interactivity.

 Comment 16: Why are AR and chat functions introduced in learning calculus?

 Response 16: The interactive AR application facilitates both human-human and human-system interactivities and assists in conceptualizing and understanding math better.

 Comment 17: Methodology

 The author may consider sub-dividing this section as below:

 i.        Study design and procedures

 ii.             The design and development of the interactive methods

 iii.            Participants

 iv.            Measurement scales

 v.             Data analysis techniques

 Response 17: We have re-arranged the sub-sections with the suggested sections such as Study design and procedures, Research Framework, Survey Design and Procedures and Data Analysis Techniques in Methodology.

 Comment 18: Study design and procedures – you should discuss the choice of quantitative research method in this research, what is the quasi-experimental design? Why is quasi-experimental design used in this research? What are the procedures to conduct this research?

 Response 18: The reason of Quantitative research method was used are added in Research Design and Participants as follows:

 Quantitative research designs include quasi-experiments, true experiments, causal-comparative research, surveys research and experiments research. Quantitative research is likely to test theories by investigating the relationships among variables [37]. Therefore, the primary research method for this study was quantitative.

 Comment 19: A discussion on the design and development of the interactive methods should be presented here.

 Response 19: A discussion on the design and development of the interactive methods is included as follows:

 Interactivity can also be categorized as human-system and human-human, as technologies are allowing both in a learning environment [3]. The framework for interactive learning application is developed based on these two types of interactions.

 Comment 20: Participants – this study uses quasi-experimental design, the participants should be assigned into control and experimental group. This information is not available. How did you assign participants into these groups? How did you select the participants for this study?

 For measurement items, what are the scales used in this study?

 Please list the sources. For the self-developed items, reliability and validity tests should be conducted and reported.

 Response 20: Additional information of participants is added in Research Design and Participants as follows:

 One of the numerous forms of quasi-experimental design is pre- and post-test research. "Quasi" refers to something that resembles experimental study. Studies evaluating a curriculum for education, a treatment system or a simulation training commonly apply pre- and post-test evaluation [33]. Since this study is to compare students' learning experiences and academic performance before and after using the AR system, a pre- and post-test quasi-experimental research method was chosen.

 Convenience sampling is a nonprobability or non-random sampling in which it is able to conveniently reach and recruit members of the target population for the study [34]. Usually, convenience samples of university students are used in academic surveys [35]. Since the respondents were students from a Malaysian university taking Calculus subject, a non-probability convenience sampling method was used for this study.

 For measurement items, the scales used in this study are included in Survey Design and Procedures - Section B as follows:

 All the questions for the aforementioned variables were measured through 5-point Likert scale.

 For the self-developed items, reliability and validity tests were conducted and included in Internal consistent reliability, as follows:

 All the self-developed questions under human-human interaction variables satisfied the reliability as the loading is >0.6 [33]. For the three self-developed questions (LE 10, LE11, and LE12) under learning experience variable two of the questions LE11, LE12 also satisfy this criterion. 

 Comment 21: What is the structural equation modelling (SEM)?

 Why is SEM used in this research?

 How to conduct the data analysis?

 You should also discuss the data analysis for longitudinal study using SEM.

 Response 21: Structural equation modeling (SEM) combines multiple regression and factor analysis. It is employed to examine the relationships between a group of observable variables and latent concepts.

 There are two main types of the SEM. In this study, instead of using the traditional Covariance-Based-SEM (CB-SEM), Partial Least Squares-SEM (PLS-SEM). PLS-SEM is chosen to analyze the data based on its capability to handle complex models and small sample sizes.

 Data analysis using PLS-SEM was employed by defining clear research objectives and hypotheses, data preparation, structural model analysis, hypotheses testing, structural model assessment and predictive accuracy, effect size and relevance evaluation [36].

 PLS-SEM is often chosen for longitudinal studies due to its effectiveness in handling small sample sizes, which are common in these studies, especially when compared to cross-sectional studies [36].

 Comment 22: Results

 As the measurement items were adopted from many different sources, instrument reliability and validity tests using Exploratory Factor Analysis (EFA) and Confirmatory Factor Analysis (CFA) should be conducted and reported.

 Response 22: CB-SEM and PLS-SEM are two widely accepted second generation data analysis approaches. PLS-SEM is used to analyze the data as the objective of the study is to confirm the hypotheses to explain the relationship between the exogenous and endogenous constructs for the following reasons [39]:

 PLS-SEM can be applied for exploratory research—when “theory is less developed”.

 PLS-SEM can handle complex cause-effect structural models.

 Additionally, data characteristics, such as small sample size and non-normal data, can be some of the reasons to choose PLS-SEM.

 Comment 23: For self-developed items, procedures to establish instrument reliability and validity should be presented.

 Response 23: For the self-developed items, reliability and validity tests were conducted and included in Internal consistency reliability, as follows:

 All the self-developed questions under human-human interaction variables satisfied the reliability as the loading is >0.6 [33]. For the three self-developed questions (LE 10, LE11, and LE12) under learning experience variable two of the questions LE11, LE12 also satisfy this criterion.  

 Comment 24: The result section needs to do a major revision with appropriate analyses. Please include relevant Table(s) with appropriate details such as paired t test results, significant value, etc. for each of the test conducted.

 Response 24: Tables 8 to 13 are included to report the results of paired t test results, significant value and other tests conducted.

 Comment 25: Discussions

 The author(s) should discuss/relate/compare the findings with prior relevant studies. Please also explain how the results in this study relate to previous findings, whether in support, contradiction, or simply as added data. The discussion should be aligned with the research objectives.

 Response 25: The discussion was rewritten with comparisons from previous studies and aligned with the research objectives as follows:

 The study aims to develop an augmented reality application that promotes interaction and spatial visualization for learning calculus and evaluates the effect of the interactive learning system on the performance of learning. Human-human interaction is evaluated as part of the chat function of application and human-system interaction is analysed as part of augmented reality implementation through mobile application.

 The first hypothesis was accepted as human-system interactivity positively affected the learning experience factor. As this study developed an augmented reality application for learning calculus, here human-system interaction is related to the interactivity promoted by the augmented reality application. Studies implementing augmented reality in improving learning experience founds that the didactic experience of this technology make students more engaged and hence providing an enriched learning experience [12, 14, 16, 21, 27]. This study has also found that among the human-system interaction all students univocally cancelled out disagreement that it provided them with real world 3D object feelings. The study using similar questionnaire have also found that it has significant impact on the learning experience in general and motivation in particular [39]. So, the result of this study is aligned with the prior relevant studies.

 The second hypothesis result found that human-human interaction significantly influences learning experience. The implication from this hypothesis acceptance indicate that learning experience is shaped by human-human interaction which can comes from student-teacher or student-student. In the study design both student-teacher and student-student interaction facilities were provided via application chat function. So, it can be said that both type of human-human interactions are associated positively with learning experience. This result is in line with the findings where both types of interaction led to learning satisfaction [47]. Another study found that human-human interactivity increases learning engagement [1, 2, 3]. In addition to that another study has also claimed that learning confidence as part of learning experience has also increased through interaction with peers and teachers [1]. From the findings of these research, it can be concluded that the result of human-human interaction affecting learning experience is in line with existing research.

 The third hypothesis of the study was tested by using paired sample “t-test” where learning performance’ means were compared from ‘pre-test’ to ‘post-test scenario. The result shows that there is significant relation between the score and “post-test” mean is higher than the ‘pre-test’ one. Other studies have also found that learning experience as result of human-system and human-human interactivity increase learning performance [3, 15, 47]. So, it can be claimed that in terms of hypothesis acceptance, this study is in line with existing literature.

 Comment 26: Additional comments:

1. Page 5, figure 1. Research design - Causal not casual relationship
2. The development of research hypotheses is missing.
3. Did not justify the adopted questionnaire.
4. Did not specify the questions for the quiz. Are the same or different sets of questions used for pre and post? How many questions?
5. Did not mention if measurement model and structural model were assessed based on data collected in the first phase or the third phase?
6. Ensure consistency in reporting the number of decimals throughout the manuscript (including in Tables)

Response 26:

1. In Figure 1, Casual Relationship is changed to Causal Relationship.
2. The development of research hypotheses is included in Section Methods - Research Framework. 
3. Justifications were added in Survey Design and Procedures - Section B.
4. Additional information for the quiz is included as follows:

The quiz included five True/False and one formative questions on solid of revolution chapter. The quiz was used in pre- and post-test evaluation for measuring learning performance factor. To avoid question biases, the same questions were used. Order of true-false questions were changed to make sure students did not just follow the similar pattern of answer from the pre-test.

 5. Phase three is included in Methods - Data Analysis Techniques as follows: The structural model was analyzed based on the data collected in phase three from post-test.

 6. Done consistency in reporting the number of decimals.

 Comment 27: Overall:

 This paper requires a significant revision to make the best of this and present the work in a clear and well-structured way, particularly on the literature review, the methodology, the research design and procedures used in this study, measurement instruments and data analysis techniques. Additionally, the discussion section is not exhaustive to provide good attempt to explain many of the study results. In fact, the discussion is not aligned with the research objectives.

 Response 27: We have revised the manuscript carefully based on all the comments and issues raised.
